# Adsorption and Removal of 2,4,6-Trinitrotoluene by a Glycoluril-Derived Molecular-Clip-Based Supramolecular Organic Framework

**DOI:** 10.3390/molecules29245822

**Published:** 2024-12-10

**Authors:** Yuezhou Liu, Shu Zeng, Xiaokai He, Yang Wu, Yang Liu, Yinglei Wang

**Affiliations:** 1 Xi’an Modern Chemistry Research Institute, Xi’an 710065, China; 21637032@zju.edu.cn (Y.L.); neu_hxk@163.com (X.H.); wuy_204@163.com (Y.W.);; 2 State Key Laboratory of Fluorine & Nitrogen Chemicals, Xi’an 710065, China

**Keywords:** 2,4,6-trinitrotoluene, energetic compounds, molecular clip, supramolecular organic framework, adsorption

## Abstract

A glycoluril-derived molecular-clip-based supramolecular organic framework (clip-SOF) with intrinsic porosity was prepared. The clip-SOF was used for the adsorption and removal of 2,4,6-trinitrotoluene (TNT) driven by noncovalent interactions. The efficiency of TNT removal by clip-SOFs is up to 88.5% in adsorption equilibrium, and the TNT adsorption capacity of clip-SOFs is about 40.2 mg/g at 25.0 °C. Clip-SOFs have good reusability, exhibiting almost no loss in performance in ten consecutive recycling tests. This work not only provides a new method for adsorbing energetic materials, but also promotes the application of supramolecular hosts in crystal engineering.

## 1. Introduction

2,4,6-Trinitrotoluene (TNT) is one of the most widely used aromatic explosives in the field of national defense and the military industry, due to its explosive properties and low sensitivity to external stimuli [1,2]. As a Group C human carcinogen, TNT has toxic influences on the ecological environment and human health [3,4]. Therefore, it is important to realize TNT removal to mitigate concerns for human health, environmental protection and public security. In the last few decades, numerous methods have been proposed and developed for the removal of TNT, such as adsorption [5,6,7,8,9], oxidation [10,11], reduction [12,13] and photo-degradation [14]. Among these treatment methods, adsorption has been regarded as a promising approach by the virtue of its easy operation, high efficiency and ability to decrease secondary pollution [15,16]. However, most of the reported adsorbents, such as activated carbon [6,15], porous organic materials [5,17,18], porous silica [19,20] and grapheme [21], suffer from a low adsorption capacity. Therefore, it is still a challenge to develop new adsorbents for TNT with a high adsorption efficiency.

Supramolecular organic frameworks (SOFs) have been recognized as a class of promising porous organic materials, which are generally constructed from functional organic modules assembled via noncovalent interactions, such as hydrogen bonds, host–guest interactions, π−π stacking and van der Waals interactions [22,23,24,25,26,27,28,29,30,31,32,33]. Due to the porous structure and soft and dynamic nature of supramolecular interactions, and the easy functionalization of their modularity, SOFs show excellent performance in absorption and separation, catalysts, sensors, and so on [34,35,36,37,38,39,40]. In particular, supramolecular host-based SOFs with intrinsic porosity have attracted great interest from scientists recently [41,42,43,44,45,46]. For example, Yang and colleagues reported on a perhydroxyl-pillar[5]arene-based SOF with a honeycomb-like structure, permanent porosity and high thermal stability, which exhibited a high affinity for CO_2_ at ambient conditions [45]. Huang and colleagues constructed a naphthalene-containing pillar[5]arene-based SOF material, which was applied for the efficient removal of adiponitrile from water, driven by host−guest complexation between pillar[5]arene and adiponitrile [46].

Molecular clips with open and adjustable cavities have attracted growing attention from material chemists and supramolecular chemists [47,48]. Their rigid structures, extensive host−guest recognition capabilities and easy functionalization properties endow molecular clips with wide applications in crystal engineering, biomedicine, sensors, supramolecular polymers, and so on [49,50,51,52,53,54]. Here, we will claim that the challenge of removing TNT pollutants can be tackled using a SOF material constructed by a glycoluril-derived molecular clip with fine-tuned functionality.

## 2. Results and Discussion

**Figure 1:**
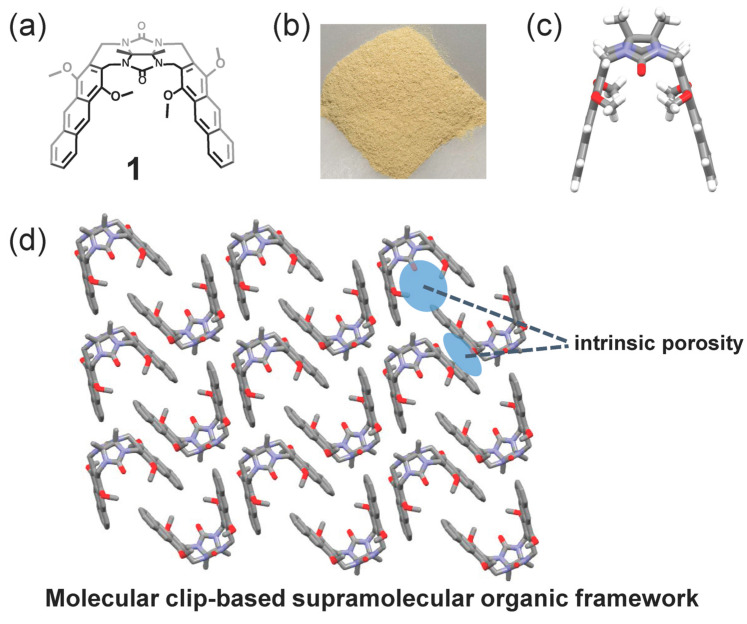
(**a**) Chemical structure of **1**; (**b**) photo of crystalline solid of **1**; (**c**) single-crystal structure of **1** based on crystallization in chloroform/*n*-hexane (*v*:*v* = 1:3, a chloroform molecule is omitted for clarity); (**d**) packing structure of **1** based on crystallization in chloroform/*n*-hexane (*v*:*v* = 1:3, chloroform molecules and hydrogen atoms are omitted for clarity).

As shown in Figure 1a, a glycoluril-derived molecular clip **1** with two anthracene groups as “arms” was prepared according to our previous work [54]. Due to the rigidity and good crystallization ability of anthracene rings, faint yellow crystals of **1** (Figure 1b) easily formed by slow evaporation during cooling of a hot saturated chloroform/*n*-hexane (*v*:*v* = 1:3) solution within 12 h, and the crystal yield was up to 92%. The crystal structure of **1** indicated that **1** possesses a clip-type structure with an open cavity, and four methoxyl groups on anthracene rings are all oriented inside the cavity (Figure 1c) [55]. Due to the unique structural features of **1** and its intermolecular noncovalent interactions, molecular-clip-based SOFs (clip-SOFs) with intrinsic porosity were obtained (Figure 1d). The clip-SOFs are π-electronic-rich frameworks which are considered to be effective adsorbents for electron-deficient adsorbate TNT, which is a highly electron-deficient nitrated compound, and often acts as a good electron acceptor [56]. Inspired by these investigations, we inferred that clip-SOFs could serve as an effective tool for realizing the adsorption of TNT via noncovalent interactions.

**Figure 2:**
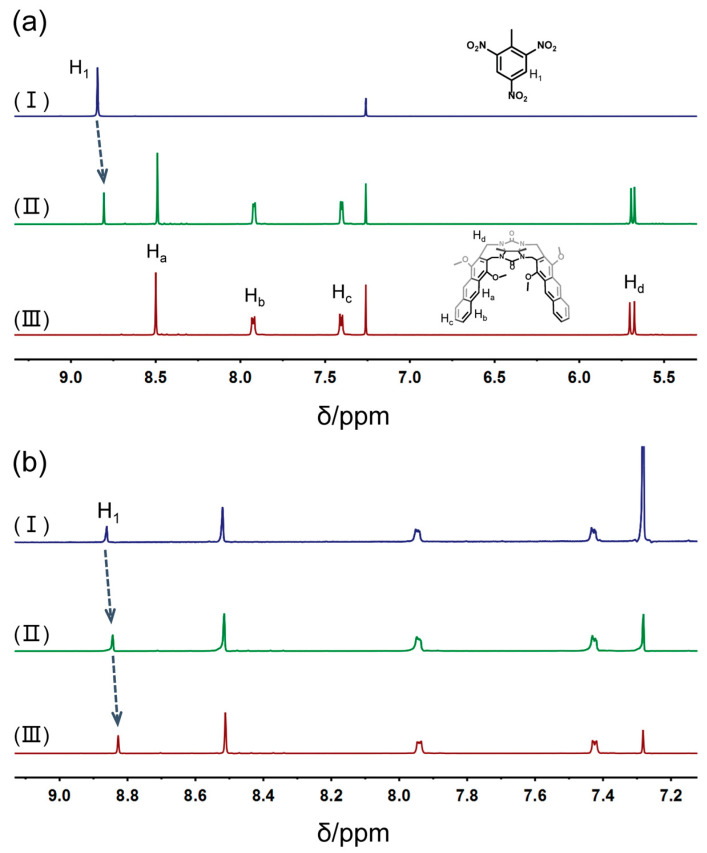
(**a**) Partial ^1^H NMR spectra (600 MHz, CDCl_3_, 298 K): (I) TNT (5.00 mM); (II) **1**•TNT (5.00 mM); (III) **1** (5.00 mM). (**b**) Partial ^1^H NMR spectra (600 MHz, CDCl_3_, 298 K) of **1**•TNT at different concentrations: (I) 1.00 mM; (II) 5.00 mM; (III) 10.0 mM.

Outstanding noncovalent interactions between **1** and TNT are a requirement for the adsorption of TNT by clip-SOFs. First, an attempt to study the interactions between **1** and TNT was carried out by ^1^H NMR characterization in CDCl_3_. As shown in Figure 2a, an obvious chemical shift of the protons on TNT was observed after mixing **1** and TNT in CDCl_3_. The signal of protons (H_1_) on TNT shifted upfield, indicating the existence of noncovalent interactions between **1** and TNT in chloroform. Moreover, the stoichiometry and association constant between **1** and TNT were determined by ^1^H NMR titration experiments (Figure S1). The *K*_a_ value was estimated to be 20.4 ± 0.7 M^−1^ for **1**•TNT in a 1:1 complexation mode by a non-linear fitting method (Figures S2 and S3).

**Figure 3:**
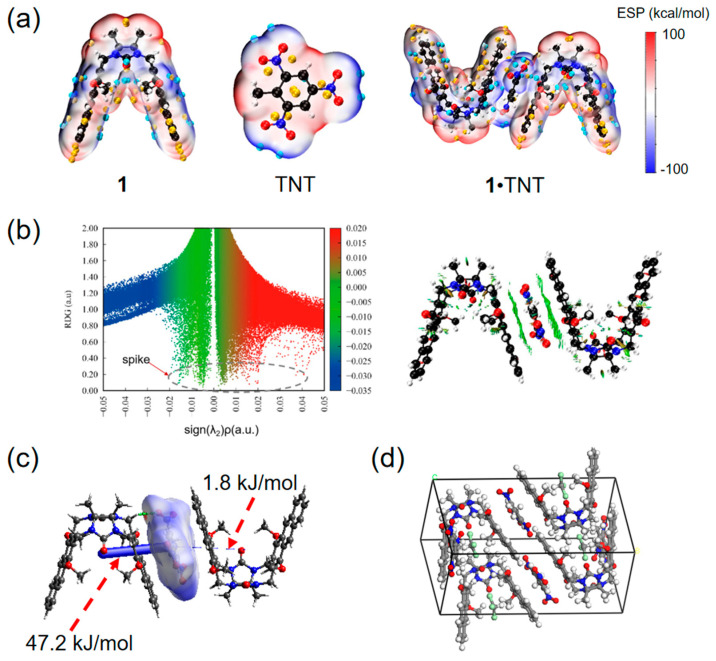
(**a**) Molecular surface electrostatic potential map of **1**, TNT and **1**•TNT; (**b**) RDG scatter distribution diagram of the interaction between **1** and TNT; (**c**) Hirshfeld surface analysis and energy framework diagram of **1**•TNT; (**d**) optimized structure of the **1**•TNT, indicating a 1:1 complexation mode between molecular clip **1** and TNT.

As shown in Figure 3a, the electrostatic potential of the surfaces of molecular clip **1**, TNT and **1**•TNT were computed. The distributions of the electrostatic potential extremum points for the individual molecular clip and TNT were observed, revealing that both molecular surfaces exhibited alternating positive and negative electrostatic potentials, which facilitated tight intermolecular binding. In Figure 3b, distinct spikes are noticeable in both the red and green regions of the RDG scatter plot, suggesting the presence of π-π interactions and intermolecular hydrogen bonds between the molecular clip and TNT. In addition, intermolecular binding energy was also computed. As shown in Figure 3c, the binding energies between TNT and the two adjacent molecular clips are 47.2 and 1.8 kJ/mol, respectively. Furthermore, from the result of the optimized structure of **1**•TNT, a 1:1 complexation mode between molecular clip **1** and TNT was also obtained.

To gain an insight into the self-assembly behavior of **1**•TNT in solution, concentration-dependent ^1^H NMR spectra of **1**•TNT were performed. As shown in Figure 2b, the signal of protons (H_1_) on TNT shifted upfield as the concentration of **1**•TNT increased from 1.00 mM to 10.0 mM, further indicating that **1** and TNT self-assembled into supramolecular aggregates.

**Figure 4:**
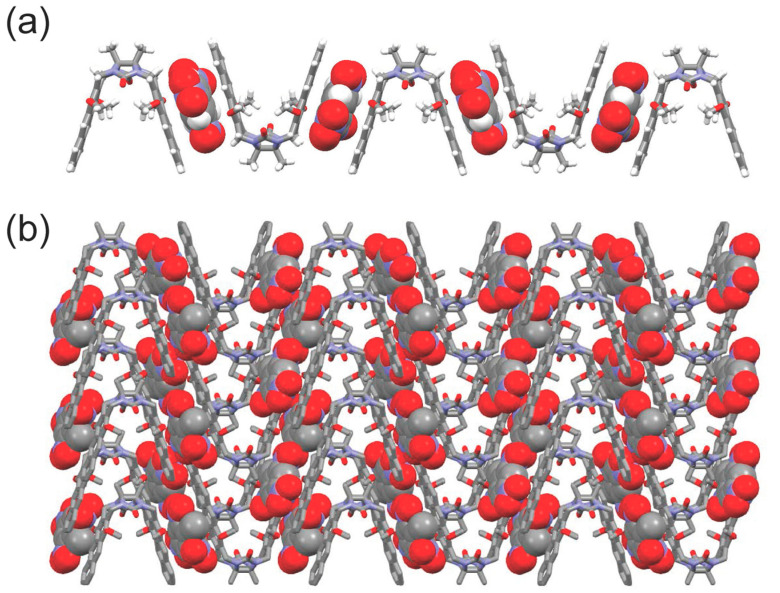
(**a**) Crystal structure of zig-zag supramolecular complex obtained by crystallization of **1**•TNT in chloroform/*n*-hexane (*v*:*v* = 1:1, chloroform molecules are omitted for clarity). (**b**) Packing structure of **1**•TNT (chloroform molecules and hydrogen atoms on **1** are omitted for clarity).

Once the supramolecular interactions between **1** and TNT were established in solution, the self-assembly behavior of **1**•TNT in its solid state was also examined. Fortunately, red single crystals of the **1**•TNT complex were obtained by the slow evaporation of a solution of **1** and TNT in chloroform/*n*-hexane (*v*:*v* = 1:1). The crystal structure of **1**•TNT was determined by single-crystal X-ray diffraction. As shown in Figures S4 and S5, a chloroform molecule is located in the cavity of **1** and a TNT molecule is inserted between two anthracene groups in the solid state. The distance between the two furthest hydrogen atoms on the two anthracene edges is about 10.972 Å. It is worth mentioning that the methoxyl groups on the anthracene rings were all oriented inside the cavity of **1**, which is architecturally consistent with clip-SOFs. Moreover, the centroid–centroid distances between the anthracene rings and a TNT molecule are 3.595 and 3.468 Å, and the dihedral angles between anthracene ring planes and a TNT ring plane are 0.84 and 2.66°. The H···O distance for the C−H···O interaction between the hydrogen atom on the methylene bridge of **1,** and the nitro oxygen atom on TNT, is 2.591 Å. Due to the synergistic effects of noncovalent interactions and hydrogen-bonding interactions between **1** and TNT, a zig-zag supramolecular complex was obtained in the solid state (Figure 4a). Furthermore, X-ray crystallographic analysis revealed that the zig-zag supramolecular complex was stabilized by C-H···π interactions with H···π-plane distances of 2.786 and 2.725 Å, and by C−H···O interactions with H···O distances of 2.619, 2.628 and 2.449 Å (Figure S6), leading to the formation of a cross-linked supramolecular structure in the solid state (Figure 4b). These results greatly support the formation of a complex between **1** and TNT in solution and in a solid state, providing the possibility of absorbing TNT using clip-SOFs.

Furthermore, the architectural stability and porosity of clip-SOFs were confirmed by measuring the N_2_ gas adsorption–desorption isotherm. The measurement of the isotherm was conducted at 78 K from 7 to 745 mmHg (Figure S7). The Brunauer–Emmett–Teller (BET) model was applied to the N_2_ gas adsorption isotherm, which resulted in an apparent surface area of S_BET_ = 73.88 m^2^/g. Furthermore, the corresponding Barrett–Joyner–Halenda (BJH) analyses suggested a predominant pore diameter distribution of 1.58 nm, with a micropore contribution of 62.03 m^2^/g, equaling 84.0% of the total surface area. The cumulative pore volume from the BJH calculations was determined to be 0.27 cm^3^/g (Figure S8). These results matched the micropore dimension expected from the single-crystal structure of clip-SOFs. Moreover, the thermal stability of clip-SOFs was characterized using thermogravimetric analysis (TGA), and the clip-SOFs showed good stability up to 179 °C (Figure S9).

**Figure 5:**
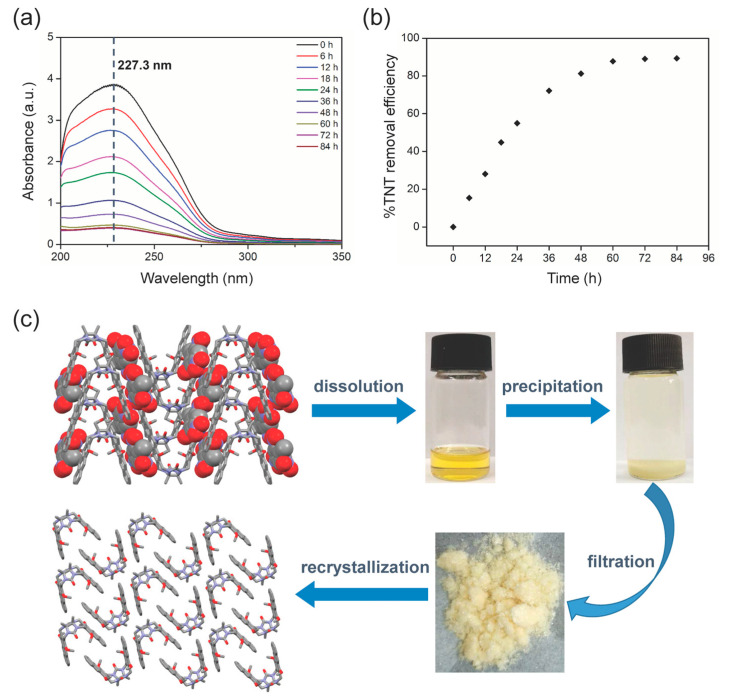
(**a**) UV–vis spectra recorded as a function of contact times of TNT (0.20 mmol/L) with clip-SOFs (1.00 mg/mL) in CH_3_OH; (**b**) time-dependent adsorption of TNT (0.20 mmol/L) by clip-SOFs (1.00 mg/mL) in CH_3_OH; (**c**) schematic illustration of the recycling of clip-SOFs.

Subsequently, the clip-SOFs were used for the efficient adsorption and removal of TNT by solid adsorption experiments at room temperature (25.0 °C) in CH_3_OH. Firstly, quantitative UV−vis spectroscopy experiments were used to investigate the TNT adsorption behavior of clip-SOFs. As shown in Figure 5a, after the immersion of clip-SOFs (50.0 mg) in a methanol solution of TNT (0.20 mmol/L, 50.0 mL), the characteristic absorbance of TNT around 227.3 nm gradually decreased over time. An adsorption equilibrium was achieved after a contact time of 72 h, and the color of the clip-SOFs changed from faint yellow to red (Figure S10), indicating the adsorption of TNT by clip-SOFs. Specifically, the efficiency of TNT removal by clip-SOFs was up to 88.5% in adsorption equilibrium (Figure 5b). The TNT adsorption capacity of clip-SOFs is about 40.2 mg/g, which is better than that of active carbon (10.40 mg/g) [57] and almost identical to that of bamboo charcoal (43.38 mg/g) [58]. Besides, it is well known that noncovalent interactions are quite sensitive to temperature. The temperature-variant NMR experiments of **1**•TNT showed that the signal of protons (H_1_) on TNT shifted downfield along with an increase in temperature from 25.0 °C to 45.0 °C (Figure S11), which indicated that host–guest interactions decreased. Moreover, temperature-dependent adsorption experiments were also carried out, and along with an increase in temperature from 25.0 °C to 45.0 °C, the TNT adsorption capacity of clip-SOFs clearly decreased from 40.2 mg/g to 22.9 mg/g (Figure S12).

Adsorbent materials with excellent reusability are always attractive. In contrast to the energy consumption and degradative regeneration processes of many traditional adsorbents^14^, clip-SOFs exhibited good recyclability in their use of a simple and energy-saving regeneration procedure. As shown in Figure 5c, red TNT-adsorbed clip-SOFs were dissolved in methylene chloride, and a faint yellow precipitate of molecular clip **1** was formed by adding methanol into the solution. The resulting precipitate was collected by filtration and dried under a vacuum. The clip-SOFs were regained by evaporation during the cooling of hot saturated chloroform/*n*-hexane (*v*:*v* = 1:3), and the recovery rate of the clip-SOFs was up to 85%, calculated using a weighing method. In addition, we performed ten consecutive recycling tests, yet clip-SOFs exhibited almost no loss in performance (Figure S13).

## 3. Materials and Methods

### 3.1. Reagents and Instruments

All the reagents were commercially available and used as supplied, without further purification. Compound **1** was prepared according to previous work [54]. ^1^H NMR spectra were recorded with an Agilent 600 MHz DirectDrive2, with the use of the deuterated solvent as the lock and the residual solvent or TMS as the internal reference. UV–vis spectra were taken on a PerkinElmer Lambda 35 UV–vis spectrophotometer. Single-crystal X-ray data sets were obtained using an Oxford Diffraction Xcalibur Atlas Gemini captra instrument, Oxford, UK. Thermogravimetric analysis (TGA) was carried out using a Q5000IR analyzer (TA instruments, Seoul, Republic of Korea) with an automated vertical overhead thermobalance. The samples were heated at a rate of 10 °C/min using N_2_ as the protective gas. An N_2_ isotherm was generated by incremental exposure to ultra-high-purity nitrogen up to 1.0 atm in a liquid nitrogen bath (78.0 K), and surface parameters were determined using the BET adsorption models included in the instrument software (BELSORP-Max, BEL Japan, Inc., Toyonaka, Japan).

### 3.2. Association Constant and Stoichiometry Determination for the Complexation Between Molecular Clip **1** and TNT

To determine the association constant and stoichiometry for the complexation between **1** and TNT, ^1^H NMR titration was performed with solutions which had a constant concentration of TNT (1.00 mM) and varying concentrations of the molecular clip **1**. By a non-linear curve-fitting method, the association constant (*K*_a_) was determined. By a mole ratio plot, 1:1 stoichiometry was obtained for the complexation between **1** and TNT.

The non-linear curve-fitting was based on the following equation [54].

Δ*δ* = (Δ*δ*_∞_/[G]_0_)(0.5[H]_0_ + 0.5([G]_0_ + 1/*K*_a_) − (0.5([H]_0_^2^ + (2[H]_0_(1/*K*_a_ − [G]_0_)) + (1/*K*_a_ + [G]_0_)^2^)^0.5^))

where Δ*δ* is the chemical shift change of H_1_ on TNT, Δ*δ*_∞_ is the chemical shift change of H_1_ when TNT is completely complexed, [H]_0_ is the initial concentration of the molecular clip **1**, and [G]_0_ is the fixed initial concentration of TNT.

### 3.3. Calculation Methods

#### 3.3.1. Molecular Surface Electrostatic Potential

Single-point energy calculations were performed using Gaussian 16 with the B3LYP/6-31+G(d, p) method. The molecular electrostatic potential was mapped to the 0.001 a.u. electron density isosurface and visualized with Multiwfn 3.8 [8,59,60].

#### 3.3.2. Intermolecular Binding Energy

Cluster structures, consisting of the central molecule and adjacent molecules within a 3.8 Å radius, were generated using CrystalExplorer 17 software. The intermolecular interaction energies were calculated using the B3LYP/6-31G(d, p) method [61,62].

#### 3.3.3. Optimize the Crystal Structures

Using the CASTEP module in Materials Studio, the calculation task was set to geometric optimization, with the calculation precision quality set to Fine [63].

### 3.4. Single Crystals Preparation Method of ***1***•TNT

**1** (10.0 mg) and TNT (5.00 mg) were dissolved in chloroform/*n*-hexane (*v*:*v* = 1:1), and the solution was allowed to evaporate slowly at room temperature, to obtain red crystals within 12 h. Following careful screening, a **1**•TNT single crystal measuring 0.1 × 0.08 × 0.06 mm^3^ was selected to ensure the acquisition of high-quality diffraction data during the X-ray diffraction analysis.

### 3.5. TNT Adsorption and Removal Experiments

TNT adsorption and removal experiments were performed at room temperature (25.0 °C) in CH_3_OH. The clip-SOFs (50.0 mg) were washed with H_2_O for 5 min and then filtered on Whatman filter paper. Then, the solid was transferred to a 100 mL round-bottomed flask. A TNT stock CH_3_OH solution (0.20 mmol/L, 50.0 mL) was added to the round-bottomed flask. The mixture was allowed to stand at room temperature, and the clear supernatant was characterized by UV–vis spectra at certain intervals.

The efficiency of TNT removal by the clip-SOFs was determined by the following equation:

%TNT removal efficiency = (*C*_0_ − *C*_t_)/*C*_0_ × 100%

where *C*_0_ (mM) and *C*_t_ (mM) are the initial and residual concentrations of the TNT in the stock solution, respectively.

### 3.6. Clip-SOF Regeneration Experiments

Clip-SOF regeneration experiments were performed via the following method. First, the red TNT-adsorbed clip-SOFs were dissolved in methylene chloride. Then, methanol was added to the solution, and a faint yellow precipitate formed. The precipitate of molecular clip **1** was collected by filtration. Finally, the clip-SOFs were regained by slow evaporation during cooling of hot saturated chloroform/*n*-hexane (*v*:*v* = 1:3) to obtain a yellow precipitate, and the recovery rate of clip-SOFs was up to 85%, calculated using a weighing method.

## 4. Conclusions

In summary, we presented a new class of clip-SOFs with intrinsic porosity, which showed good TNT adsorption capacity. The clip-SOFs were easily obtained in a high yield by crystallization in chloroform/*n*-hexane (*v*:*v* = 1:3). The architectural stability and porosity of the clip-SOFs were confirmed by measuring the N_2_ gas adsorption–desorption isotherm, and the apparent surface area of the clip-SOFs was 73.88 m^2^/g, with 84.0% micropore contribution. The electron-rich molecular clip **1** was capable of complexing with TNT to form a supramolecular complex with 1:1 stoichiometry, driven by noncovalent interactions in chloroform. The self-assembly behaviors of **1**•TNT were characterized in detail, both in solution and in a solid state. TNT adsorption and removal experiments indicated that the efficiency of TNT removal by clip-SOFs was up to 88.5% in adsorption equilibrium, and the TNT adsorption capacity of clip-SOFs was about 40.2 mg/g, which is better than that of active carbon and almost identical to that of bamboo charcoal. Moreover, the clip-SOFs could be regenerated using a mild procedure, and showed no loss in performance in ten consecutive recycling tests. However, the adsorption equilibrium time was very long (72 h), and the absorption efficiency was sensitive to changes in temperature, due to the weak interactions between the molecular clip and TNT. This work not only provides a new TNT adsorption approach with the use of porous π-electronic-rich clip-SOFs, but also accelerated the application of supramolecular hosts in crystal engineering.

## Data Availability

The data presented in this study are available in the paper.

## Supplementary Materials

The following materials are available online: https://www.mdpi.com/article/10.3390/molecules29245822/s1. Figure S1: Partial ^1^H NMR spectra of TNT at a concentration of 1.00 mM, upon addition of various concentrations of the molecular clip **1**; Figure S2: Mole ratio plot for TNT and **1**, indicating a 1:1 stoichiometry; Figure S3: The chemical shift changes of H_1_ on TNT upon addition of **1**; Figures S4–S6: Single-crystal structure of **1**•TNT; Figure S7: Experimental N_2_ adsorption–desorption isotherms at 78 K, measuring the porosity of clip-SOFs; Figure S8: The cumulative pore area (a) and pore volume (b) of clip-SOFs obtained by Barrett–Joyner–Halenda (BJH) analyses, indicating the microporous structure of clip-SOFs; Figure S9: Thermogravimetric analysis of clip-SOFs; Figure S10: Colour changes of clip-SOFs before and after the adsorption of TNT; Figure S11: Partial ^1^H NMR spectra (600 MHz, CDCl_3_, 5.00 mM) of **1**•TNT at different temperatures; Figure S12: Temperature-dependent adsorption of TNT (0.20 mmol/L) by clip-SOFs (1.00 mg/mL) in CH_3_OH; Figure S13: The regeneration cycles of clip-SOFs after the adsorption of TNT; Table S1: Experimental single-crystal X-ray data for **1**•TNT; Table S2: TNT adsorption capacity of clip-SOFs along with changes in temperature.

## References

[1] Wang S., Wang Q., Feng X., Wang B., Yang L. (2017). Explosives in the Cage: Metal–Organic Frameworks for High-Energy Materials Sensing and Desensitization. Adv. Mater..

[2] Feng L., Li H., Qu Y., Lü C. (2012). Detection of TNT Based on Conjugated Polymer Encapsulated in Mesoporous Silica Nanoparticles through FRET. Chem. Commun..

[3] Zhang R., Zhang C., Zheng F., Li X., Sun C.-L., Chen W. (2018). Nitrogen and Sulfur Co-Doped Graphene Nanoribbons: A Novel Metal-Free Catalyst for High Performance Electrochemical Detection of 2, 4, 6-Trinitrotoluene (TNT). Carbon.

[4] Ariyarathna T., Vlahos P., Tobias C., Smith R. (2016). Sorption Kinetics of TNT and RDX in Anaerobic Freshwater and Marine Sediments: Batch Studies. Enviro. Toxic Chem..

[5] Wang Y., Zhang L., Yang L., Ma Y., Chang G. (2019). A Recyclable Indole-Based Polymer for Trinitrotoluene Adsorption via the Synergistic Effect of Dipole–π and donor–Acceptor Interactions. Polym. Chem..

[6] Marinović V., Ristić M., Dostanić M. (2005). Dynamic Adsorption of Trinitrotoluene on Granular Activated Carbon. J. Hazard. Mater..

[7] Yao Y., Xue M., Chen J., Zhang M., Huang F. (2012). An Amphiphilic Pillar[5]Arene: Synthesis, Controllable Self-Assembly in Water, and Application in Calcein Release and TNT Adsorption. J. Am. Chem. Soc..

[8] Fu D., Zhang Y., Lv F., Chu P.K., Shang J. (2012). Removal of Organic Materials from TNT Red Water by Bamboo Charcoal Adsorption. Chem. Eng. J..

[9] Zhong C., He P., Yuan R., Sun Y., Deng H., Zhang T., Liang S., Kang B., Chang G., Xu Y. (2023). Hydrophobic Porous Polymer Containing Isoxazoline and Siloxane Groups for 2,4,6-Trinitrotoluene (TNT) Adsorption via Synergistic Effect of Lewis Acid-Base, Dipole-π, and π-π Interactions. J. Non-Cryst. Solids.

[10] Ayoub K., Nélieu S., Van Hullebusch E.D., Maia-Grondard A., Cassir M., Bermond A. (2011). TNT Oxidation by Fenton Reaction: Reagent Ratio Effect on Kinetics and Early Stage Degradation Pathways. Chem. Eng. J..

[11] Matta R., Hanna K., Kone T., Chiron S. (2008). Oxidation of 2,4,6-Trinitrotoluene in the Presence of Different Iron-Bearing Minerals at Neutral pH. Chem. Eng. J..

[12] Casey M.C., Cliffel D.E. (2015). Surface Adsorption and Electrochemical Reduction of 2,4,6-Trinitrotoluene on Vanadium Dioxide. Anal. Chem..

[13] Zhang X., Lin Y., Shan X., Chen Z. (2010). Degradation of 2,4,6-Trinitrotoluene (TNT) from Explosive Wastewater Using Nanoscale Zero-Valent Iron. Chem. Eng. J..

[14] Son H.-S., Lee S.-J., Cho I.-H., Zoh K.-D. (2004). Kinetics and Mechanism of TNT Degradation in TiO2 Photocatalysis. Chemosphere.

[15] Lee J.-W., Yang T.-H., Shim W.-G., Kwon T.-O., Moon I.-S. (2007). Equilibria and Dynamics of Liquid-Phase Trinitrotoluene Adsorption on Granular Activated Carbon: Effect of Temperature and pH. J. Hazard. Mater..

[16] Chatterjee S., Paital A.R. (2018). Functionalized Cubic Mesoporous Silica as a Non-Chemodosimetric Fluorescence Probe and Adsorbent for Selective Detection and Removal of Bisulfite Anions along with Toxic Metal Ions. Adv. Funct. Mater..

[17] Wang Y., Zhang L., Yang L., Chang G. (2020). An Indole-Based Smart Aerogel for Simultaneous Visual Detection and Removal of Trinitrotoluene in Water via Synergistic Effect of Dipole-π and donor-Acceptor Interactions. Chem. Eng. J..

[18] Zhao H., Ma X., Li Y., Du R., Zhang Z., An F., Gao B. (2015). Selective Detection of TNT Using Molecularly Imprinted Polymer Microsphere. Desalination Water Treat..

[19] An F., Gao B., Feng X. (2009). Adsorption of 2,4,6-Trinitrotoluene on a Novel Adsorption Material PEI/SiO2. J. Hazard. Mater..

[20] An F., Gao B., Feng X. (2009). Adsorption Performance and Mechanism of 2,4,6-Trinitrotoluene on a Novel Adsorption Material Polyvinylbenzyl Acid/SiO2. Appl. Surf. Sci..

[21] Bharti, Khurana I., Shaw A.K., Saxena A., Khurana J.M., Rai P.K. (2018). Removal of Trinitrotoluene with Nano Zerovalent Iron Impregnated Graphene Oxide. Water Air Soil Pollut.

[22] Tian J., Chen L., Zhang D.-W., Liu Y., Li Z.-T. (2016). Supramolecular Organic Frameworks: Engineering Periodicity in Water Through Host-Guest Chemistry. Chem. Commun..

[23] Li Z.-T., Yu S.-B., Liu Y., Tian J., Zhang D.-W. (2022). Supramolecular Organic Frameworks: Exploring Water-Soluble, Regular Nanopores for Biomedical Applications. Acc. Chem. Res..

[24] Kim H., Kim Y., Yoon M., Lim S., Park S.M., Seo G., Kim K. (2010). Highly Selective Carbon Dioxide Sorption in an Organic Molecular Porous Material. J. Am. Chem. Soc..

[25] Tian J., Ma S., Thallapally P.K., Fowler D., McGrail B.P., Atwood J.L. (2011). Cucurbit[7]Uril: An Amorphous Molecular Material for Highly Selective Carbon Dioxide Uptake. Chem. Commun..

[26] Hasell T., Schmidtmann M., Cooper A.I. (2011). Molecular Doping of Porous Organic Cages. J. Am. Chem. Soc..

[27] Hasell T., Chong S.Y., Jelfs K.E., Adams D.J., Cooper A.I. (2012). Porous Organic Cage Nanocrystals by Solution Mixing. J. Am. Chem. Soc..

[28] Sozzani P., Bracco S., Comotti A., Ferretti L., Simonutti R. (2005). Methane and Carbon Dioxide Storage in a Porous van Der Waals Crystal. Angew. Chem. Int. Ed..

[29] He Y., Xiang S., Chen B. (2011). A Microporous Hydrogen-Bonded Organic Framework for Highly Selective C_2_H_2_/C_2_H_4_ Separation at Ambient Temperature. J. Am. Chem. Soc..

[30] Mastalerz M., Oppel I.M. (2012). Rational Construction of an Extrinsic Porous Molecular Crystal with an Extraordinary High Specific Surface Area. Angew. Chem. Int. Ed..

[31] Luo X.-Z., Jia X.-J., Deng J.-H., Zhong J.-L., Liu H.-J., Wang K.-J., Zhong D.-C. (2013). A Microporous Hydrogen-Bonded Organic Framework: Exceptional Stability and Highly Selective Adsorption of Gas and Liquid. J. Am. Chem. Soc..

[32] Yamamoto A., Hamada T., Hisaki I., Miyata M., Tohnai N. (2013). Dynamically Deformable Cube-like Hydrogen-Bonding Networks in Water-Responsive Diamondoid Porous Organic Salts. Angew. Chem. Int. Ed..

[33] Li P., He Y., Guang J., Weng L., Zhao J.C.-G., Xiang S., Chen B. (2014). A Homochiral Microporous Hydrogen-Bonded Organic Framework for Highly Enantioselective Separation of Secondary Alcohols. J. Am. Chem. Soc..

[34] Patil R.S., Banerjee D., Zhang C., Thallapally P.K., Atwood J.L. (2016). Selective CO_2_ Adsorption in a Supramolecular Organic Framework. Angew. Chem. Int. Ed..

[35] Kong J., Du J., Wang J., Chen Z. (2015). Supramolecular Organic Frameworks of a Schiff Base Showing Selective Guest Adsorption. J. Appl. Crystallogr..

[36] Lü J., Perez-Krap C., Suyetin M., Alsmail N.H., Yan Y., Yang S., Lewis W., Bichoutskaia E., Tang C.C., Blake A.J. (2014). A Robust Binary Supramolecular Organic Framework (SOF) with High CO_2_ Adsorption and Selectivity. J. Am. Chem. Soc..

[37] Yu S., Qi Q., Yang B., Wang H., Zhang D., Liu Y., Li Z. (2018). Enhancing Hydrogen Generation Through Nanoconfinement of Sensitizers and Catalysts in a Homogeneous Supramolecular Organic Framework. Small.

[38] Yan M., Liu X.-B., Gao Z.-Z., Wu Y.-P., Hou J.-L., Wang H., Zhang D.-W., Liu Y., Li Z.-T. (2019). A Pore-Expanded Supramolecular Organic Framework and Its Enrichment of Photosensitizers and Catalysts for Visible-Light-Induced Hydrogen Production. Org. Chem. Front..

[39] Jiang M., Wang Y., Liu H., Yu S., Niu K.-K., Xing L.-B. (2024). Construction of a Novel Pyrene-based Two-Dimensional Supramolecular Organic Framework and the Selective Regulation of Reactive Oxygen Species for Photocatalysis. J. Mater. Chem. A.

[40] Yin C., Yan Z.-A., Yan R., Xu C., Ding B., Ji Y., Ma X. (2024). A 3D Phosphorescent Supramolecular Organic Framework in Aqueous Solution. Adv. Funct. Mater..

[41] Tedesco C., Erra L., Brunelli M., Cipolletti V., Gaeta C., Fitch A.N., Atwood J.L., Neri P. (2010). Methane Adsorption in a Supramolecular Organic Zeolite. Chem. A Eur. J.

[42] Tsue H., Ono K., Tokita S., Ishibashi K., Matsui K., Takahashi H., Miyata K., Takahashi D., Tamura R. (2011). Spontaneous and Selective CO_2_ Sorption under Ambient Conditions in Seemingly Nonporous Molecular Crystal of Azacalix[5]Arene Pentamethyl Ether. Org. Lett..

[43] Sun Y.-L., Zhou Y., Li Q.-L., Yang Y.-W. (2013). Enzyme-Responsive Supramolecular Nanovalves Crafted by Mesoporous Silica Nanoparticles and Choline-Sulfonatocalix[4]Arene [2]Pseudorotaxanes for Controlled Cargo Release. Chem. Commun..

[44] Chaix A., Mouchaham G., Shkurenko A., Hoang P., Moosa B., Bhatt P.M., Adil K., Salama K.N., Eddaoudi M., Khashab N.M. (2018). Trianglamine-Based Supramolecular Organic Framework with Permanent Intrinsic Porosity and Tunable Selectivity. J. Am. Chem. Soc..

[45] Tan L., Li H., Tao Y., Zhang S.X., Wang B., Yang Y. (2014). Pillar[5]arene-Based Supramolecular Organic Frameworks for Highly Selective CO_2_ -Capture at Ambient Conditions. Adv. Mater..

[46] Shi B., Shangguan L., Wang H., Zhu H., Xing H., Liu P., Liu Y., Liu J., Huang F. (2019). Pillar[5]Arene-Based Molecular Recognition Induced Crystal-to-Crystal Transformation and Its Application in Adsorption of Adiponitrile in Water. ACS Mater. Lett..

[47] Hardouin–Lerouge M., Hudhomme P., Sallé M. (2011). Molecular Clips and Tweezers Hosting Neutral Guests. Chem. Soc. Rev..

[48] Klärner F.-G., Kahlert B. (2003). Molecular Tweezers and Clips as Synthetic Receptors. Molecular Recognition and Dynamics in Receptor−Substrate Complexes. Acc. Chem. Res..

[49] Schrader T., Bitan G., Klärner F.-G. (2016). Molecular Tweezers for Lysine and Arginine—Powerful Inhibitors of Pathologic Protein Aggregation. Chem. Commun..

[50] Jono K., Suzuki A., Akita M., Albrecht K., Yamamoto K., Yoshizawa M. (2017). A Polyaromatic Molecular Clip That Enables the Binding of Planar, Tubular, and Dendritic Compounds. Angew. Chem. Int. Ed..

[51] Harmata M. (2004). Chiral Molecular Tweezers. Acc. Chem. Res..

[52] Wang J., Wang M., Xiang J., Cao L., Wu A., Isaacs L. (2015). Dimeric Packing of Molecular Clips Induced by Interactions between π-Systems. CrystEngComm.

[53] Shanmugaraju S., Mukherjee P.S. (2015). Self-Assembled Discrete Molecules for Sensing Nitroaromatics. Chem. A Eur. J..

[54] Liu Y., Chen P., Shi B., Jiao T., Ju H., Liu P., Huang F. (2020). Cocrystallization with a Clip-Type Molecule Catcher: A New Method to Determine Structures of Liquid Molecules. Org. Chem. Front..

[55] Deposition Numbers 1960287 (for 1) and 2219664 (for 1•TNT) Contain the Supplementary Crystallographic Data for This Paper. These Data Are Provided Free of Charge by the Joint Cambridge Crystallographic Data Centre and Fachinformationszentrum Karlsruhe Access Structures Service.

[56] Gong Y.-N., Jiang L., Lu T.-B. (2013). A Highly Stable Dynamic Fluorescent Metal–Organic Framework for Selective Sensing of Nitroaromatic Explosives. Chem. Commun..

[57] Zhang M.H., Zhao Q.L., Ye Z.F. (2011). Organic Pollutants Removal from 2,4,6-Trinitrotoluene (TNT) Red Water Using LowCost Activated Coke. J. Environ. Sci..

[58] Frisch M., Trucks G.W., Schlegel H.B., Scuseria G.E., Robb M.A., Cheeseman J.R., Scalmani G., Barone V., Petersson G.A., Nakatsuji H. (2016). Gaussian 16, Revision C. 01.

[59] Zhao Y., Truhlar D.G. (2008). The M06 Suite of Density Functionals for Main Group Thermochemistry, Thermochemical Kinetics, Noncovalent Interactions, Excited States, and Transition Elements: Two New Functionals and Systematic Testing of Four M06-Class Functionals and 12 Other Functionals. Theor. Chem. Acc..

[60] Lu T., Chen F. (2012). Multiwfn: A Multifunctional Wavefunction Analyzer. J. Comput. Chem..

[61] Spackman P.R., Turner M.J., McKinnon J.J., Wolff S.K., Grimwood D.J., Jayatilaka D., Spackman M.A. (2021). CrystalExplorer: A program for Hirshfeld surface analysis, visualization and qu-antitative analysis of molecular crystals. J. Appl. Cryst..

[62] He X., Chen C., Zhang Z., Yu T., Wen L., Cao Y., Liu Y. (2024). Molecule Empowerment and Crystal Desensitization: A Multilevel Structure-Property Analysis toward Designing High-Energy Low-Sensitivity Layered Energetic Materials. ACS Appl. Mater. Interfaces.

[63] Sun H. (1998). COMPASS: An Ab Initio Force-Field Optimized for Condensed-Phase Applications Overview with Details on Alkane and Benzene Compounds. J. Phys. Chem. B.

